# Prophylactic Soraphen A Treatment Attenuates Western Diet‐Exacerbated IMQ‐Induced Psoriasiform Inflammation

**DOI:** 10.1002/eji.70298

**Published:** 2026-10-27

**Authors:** Yu‐San Kao, Katharina S. Kommoss, Tabea Bieler, Gloria Janet Godoy, Aleksandra Lopez Krol, Fatima Boukhallouk, Felix K. F. Kommoss, Danijela Heide, Susanne H. Kirsch, Rolf Müller, Mathias Heikenwälder, Tim Sparwasser

**Affiliations:** ^1^ Institute of Medical Microbiology and Hygiene University Medical Center of the Johannes Gutenberg University Mainz Mainz Germany; ^2^ Division of Chronic Inflammation and Cancer German Cancer Research Center (DKFZ) Heidelberg Germany; ^3^ Department of Dermatology University Hospital Heidelberg Heidelberg Germany; ^4^ Fachbereich Biologie Johannes Gutenberg University Mainz Mainz Germany; ^5^ Institute of Molecular Medicine University Medical Center of the Johannes Gutenberg University Mainz Mainz Germany; ^6^ Institute of Pathology University Hospital Heidelberg Heidelberg Germany; ^7^ Helmholtz Institute for Pharmaceutical Research Saarland (HIPS) Helmholtz Centre for Infection Research (HZI) Saarland University Campus Saarbrücken Germany; ^8^ German Centre for Infection Research (DZIF) Partner Site Hannover‐Braunschweig Braunschweig Germany; ^9^ Department of Pharmacy Saarland University Saarbrücken Germany; ^10^ The M3 Research Center Institute For Interdisciplinary Research On Cancer Metabolism and Chronic Inflammation Eberhard Karls University Tübingen Medical Faculty Tübingen Germany; ^11^ Research Center for Immunotherapy (FZI) University Medical Center Johannes Gutenberg‐University Mainz Mainz Germany

**Keywords:** acetyl‐CoA carboxylase (ACC) 1, fatty acid synthesis, metabolic inflammation, psoriasis

## Abstract

Psoriasis is a T‐cell‐mediated inflammatory disease amplified by metabolic stress. Acetyl‐CoA carboxylase 1 (ACC1)‐dependent fatty acid synthesis is required for IL‐17‐producing T cells. Here, we asked whether short‐term systemic administration of soraphen A (SorA), a dual ACC1/2 inhibitor, modulates imiquimod (IMQ)‐induced psoriasiform inflammation under normal diet (ND) and Western diet (WD) conditions. Female C57BL/6J mice were fed ND or WD for 12 weeks prior to daily intraperitoneal injections of SorA or vehicle for 10 days, followed by 5 days of topical IMQ to induce psoriasiform dermatitis. Disease severity was assessed by macroscopic, histological, transcriptional, and flow cytometric readouts. Prophylactic SorA administration had no measurable effect in ND‐fed mice. In WD‐fed mice, prophylactic SorA reduced erythema, back skin thickening, and epidermal hyperplasia, whereas scaling remained unchanged. Selected histological skin readouts were numerically lower, whereas the qPCR panel showed no statistically significant differences. In skin‐draining lymph nodes, IL‐17A^+^ CD4^+^ and CD8^+^ T‐cell frequencies and numbers were significantly reduced, whereas γδT17 cells did not differ significantly. Hepatic steatosis scores were unchanged, but hepatic neutrophil infiltration was reduced. Under the present prophylactic regimen, systemic SorA administration was associated with modest attenuation of WD‐exacerbated IMQ‐induced psoriasiform inflammation. These findings support further preclinical evaluation of ACC‐targeted immunometabolic interventions in metabolically aggravated psoriasiform inflammation.

## Graphical abstract

In Western diet‐fed mice, prophylactic Soraphen A administration partially reduced erythema, back skin thickening, and epidermal hyperplasia. Hepatic steatosis scores were unchanged, but neutrophil infiltration was reduced. In skin‐draining lymph nodes, IL‐17A^+^ CD4^+^ and CD8^+^ T‐cell frequencies and numbers were significantly reduced, whereas γδT17 cells remained unchanged. Created in BioRender. Kao, Y. (2026) https://BioRender.com/e0udb71

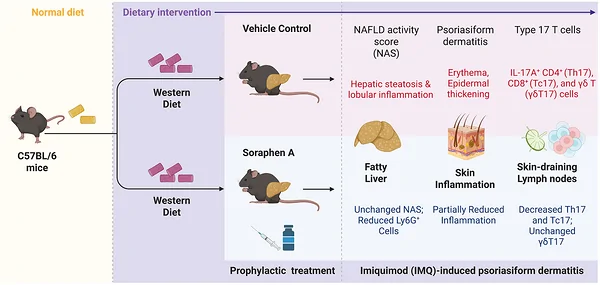

**Abbreviations**
ACC1: acetyl‐CoA carboxylase 1
ACC2: acetyl‐CoA carboxylase 2
CCL: C‐C motif chemokine ligand
CD31: platelet endothelial cell adhesion molecule‐1
CPT1: carnitine palmitoyltransferase 1
EC: endothelial cell
FAS: fatty acid synthesis
FASN: fatty acid synthase
FFPE: formalin‐fixed, paraffin‐embedded
γδT17: IL‐17‐producing gamma delta T cell
ICAM‐1: intercellular adhesion molecule 1
IL: interleukin
IMQ: imiquimod
i.p.: intraperitoneal
MASLD: metabolic dysfunction‐associated steatotic liver disease
NAFLD: nonalcoholic fatty liver disease
NAS: NAFLD activity score
ND: normal diet
PASI: psoriasis area and severity index
Rps14: ribosomal protein s14
SorA: soraphen A
Tc cell: cytotoxic T cell
Teff: effector T cells
Th cell: T helper cell
Treg: regulatory T cells
TNF‐α: tumor necrosis factor alpha
WD: Western diet

## 1 Introduction

Psoriasis is a chronic, T‐cell‐mediated inflammatory disease primarily driven by the IL‐23/IL‐17 axis, which promotes keratinocyte hyperproliferation and inflammation [1]. Beyond cutaneous inflammation, psoriasis is associated with obesity and cardiometabolic comorbidities, including cardiovascular disease, type 2 diabetes mellitus, and metabolic dysfunction‐associated steatotic liver disease (MASLD), with interorgan crosstalk potentially sustaining feed‐forward inflammatory loops [2].

Western diet (WD), a dietary pattern rich in fat and sugars and low in dietary fiber, induces dyslipidemia, hyperglycemia, and obesity, linking to the development of metabolic syndrome. WD exacerbates the psoriasiform phenotype partly by increasing IL‐17‐producing T cells in murine models [3, 4, 5]. The underlying mechanisms are not fully understood but partially involve the IL‐23/IL‐17 axis [6]. Thus, targeting metabolic dysregulation may offer new therapeutic strategies.

Metabolic reprogramming is pivotal for naive T cells to activate and differentiate into effector T cells (Teff) or regulatory T cells (Treg) [7]. Specifically, *de novo* fatty acid synthesis (FAS) is essential for the differentiation of naïve αβ T cells into T helper 17 (Th17) effector cells, but not for Treg [7, 8]. Inhibiting the rate‐limiting enzyme of FAS, acetyl‐CoA carboxylase 1 (ACC1), reduces the Th17/Treg ratio, thereby favoring an anti‐inflammatory response [7, 8, 9]. In addition to conventional Teff, innate‐like IL‐17‐producing‐γδ T (γδT17) cells, specifically dermal Vγ4^+^ γδ T cells, are selectively expanded and constitute a major source of IL‐17A in the Imiquimod (IMQ) psoriasis‐like dermatitis mouse model [10, 11, 12]. Psoriatic inflammation promotes upregulation of ACC1‐mediated FAS in γδT17 cells, supporting their IL‐17A production [13]. Further, our research group recently showed that T‐cell‐specific ACC1 deletion ameliorates psoriatic skin inflammation [13, 14]. These findings identify ACC1 inhibition as a potential therapeutic strategy in psoriasis.

ACCs catalyze the ATP‐dependent carboxylation of acetyl‐CoA to malonyl‐CoA. Cytosolic ACC1 is essential for *de novo* FAS, whereas ACC2 is a regulator of mitochondrial fatty acid oxidation [15]. Soraphen A (SorA), a myxobacterial natural product, inhibits both ACC1 and ACC2; its effects on Th17 differentiation have been shown to depend largely on ACC1 [7, 16, 17]. Additionally, ACC1 has been implicated in regulating endothelial cell (EC) migration and vascular dysfunction by controlling FAS and membrane lipid composition [18]. Therefore, we investigated systemic administration of SorA in the IMQ‐induced psoriasis‐like dermatitis model. Based on previous mechanistic work, the present study was designed as a short prophylactic in vivo proof‐of‐concept study rather than an isoform‐specific mechanistic dissection. We further asked whether WD‐associated metabolic stress unmasks SorA‐associated effects in this model.

## 2 Results

### 2.1 Prophylactic SorA Administration Partially Attenuates Psoriasiform Skin Inflammation Under Western Diet Conditions

To model diet‐exacerbated psoriatic skin inflammation, female C57BL/6J mice were maintained on a high‐fat/fructose Western diet (WD) for 12 weeks prior to prophylactic SorA administration and followed by topical IMQ application (Figure 1A). Increased body weight was observed after 12 weeks of WD, before prophylactic SorA injections (Figure 1B; Figures S1A,B and S2A). During prophylactic SorA administration before IMQ application, skin thickness changes were comparable between SorA and vehicle controls (Figure S2B). Prophylactic SorA administration ameliorated IMQ‐associated body weight loss, compared to vehicle controls (Figure 1B). SorA administration was associated with lower macroscopic psoriasiform inflammation (Figure 1C). This was quantified by reduced erythema (Figure 1D), unchanged scaling (Figure 1E), and significantly reduced back skin thickening (Figure 1F), leading to an overall reduced modified Psoriasis Area and Severity Index (PASI) Score (Figure 1G). Decreased back skin thickening was corroborated histologically (Figure 1H,I). In contrast, under normal diet (ND) conditions, mice receiving prophylactic SorA showed no differences in IMQ‐induced psoriasiform dermatitis (Figure S1C–I). SorA‐associated effects were detectable under WD but not ND conditions, suggesting that the response to this short prophylactic regimen is context‐dependent.

**FIGURE 1:**
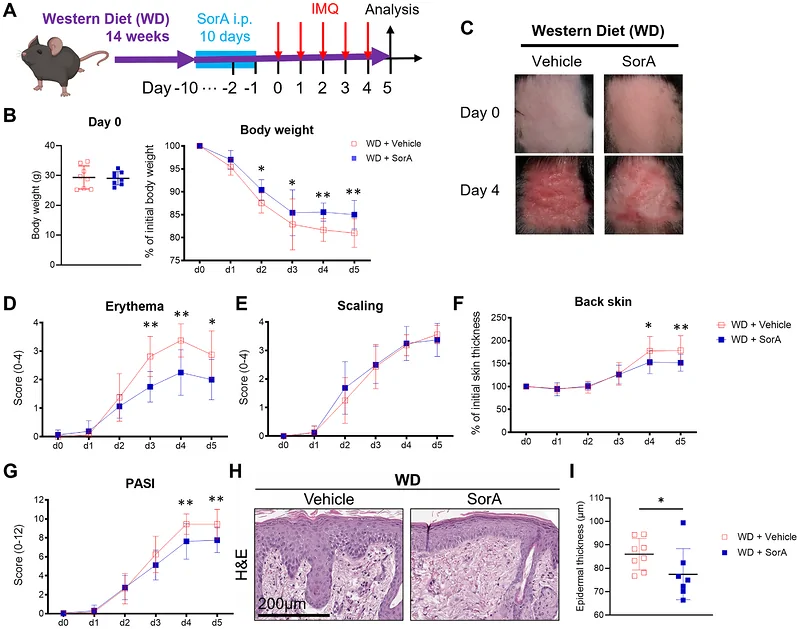
Prophylactic Soraphen A (SorA) partially attenuates clinical and histological features of Western‐diet (WD)‐exacerbated Imiquimod (IMQ)‐induced psoriasiform dermatitis. (**A**) 7‐week‐old female mice were fed WD for 12 weeks, followed by 10 daily intraperitoneal injections of the ACC inhibitor Soraphen A (SorA; 20 mg/kg/day) or vehicle, and five days of topical imiquimod (IMQ) challenge (total study duration approximately 14 weeks). (**B**) Body weight on day 0 (= start of IMQ treatment) and body‐weight change during IMQ treatment. (**C**) Representative images of back skin, clinical scores of (**D**) erythema, (**E**) scaling, and (**F**) thickness of IMQ‐treated back skin, and (**G**) composite modified psoriasis area and severity index (PASI) score (erythema, scaling, and back skin thickness) measured daily during the IMQ treatment period. Back skin thickness was normalized to day 0 of the IMQ model. (**H**) Skin sections were collected from the IMQ‐treated back on day 5 for hematoxylin and eosin (H&E) staining; representative histological sections are shown. (**I**) Epidermal thickness measurements quantified in H&E staining are shown. WD + vehicle = mice fed with WD and treated with vehicle (*n* = 8 for **B**‐**G**; n=7 for **I**), and WD + SorA = mice fed with WD and treated with SorA (*n* = 8). Error bars represent standard deviation (SD). *p*‐values were obtained by two‐way ANOVA with mixed‐effects/repeated‐measures models in (**B**–**G**) and an unpaired *t*‐test in (**I**). ns, not significant = *p* > 0.05, *p < 0.05; **p < 0.01; ***p < 0.001; ****p < 0.0001.

### 2.2 Prophylactic SorA Administration is Associated With Modest Changes in Vascular, Proliferative, and Inflammatory Skin Readouts

To further characterize the reduction in skin inflammation in SorA‐treated WD‐fed mice, we assessed markers associated with vascular activation, cellular proliferation, epithelial activation, and inflammatory signaling. Histological analysis of CD31^+^ (Platelet endothelial cell adhesion molecule‐1) microvessel density and epidermal Ki67^+^ (a nuclear marker of active proliferation) cell density staining were numerically lower under SorA conditions but did not reach statistical significance (Figure 2A–D). Using qPCR analysis, endothelial activation was evaluated by measuring *Icam1*, an endothelial adhesion molecule involved in leukocyte adhesion and transmigration [19]. Proliferative responses were assessed through *Mki67* (encoding Ki67) [20], *Pcna*, a key regulator of DNA replication and repair [21], and *Ccnb1* (Cyclin B1), which regulates the G2/M transition of the cell cycle [22]. We also examined *Krt17* (Keratin 17), an intermediate filament protein associated with epithelial activation, proliferation, and tissue repair that is aberrantly expressed in psoriatic skin lesions [23]. In addition, inflammatory skin responses were evaluated by measuring transcripts of *Il17a* and *Il17f*, which encode pro‐inflammatory cytokines that promote neutrophil recruitment and amplify inflammatory responses, particularly in psoriasis [24], *Ifnb1*, which encodes a type I interferon implicated in skin inflammation and produced by keratinocytes [25], *Il18*, which encodes a pro‐inflammatory cytokine upregulated in psoriatic lesions that promotes IFN‐γ production and innate immune activation [26, 27], and *Ccl20*, which encodes a chemokine that recruits CCR6‐expressing immune cells, including Th17 cells and dendritic cells, to inflammatory sites [24]. Transcript levels of these genes showed no statistically significant differences (Figure 2E). Ly6G^+^ cell density in skin was numerically lower but did not differ significantly (Figure 2F‐G). Neutrophil‐associated transcripts (*Cxcr4*, *Elane*, *Ctsg*, *Tnfaip3*, *Cebpe*) showed no significant change (Figure 2H). Together, these data are consistent with a modest reduction in epidermal hyperplasia and skin inflammation under prophylactic SorA treatment in WD‐fed mice.

**FIGURE 2:**
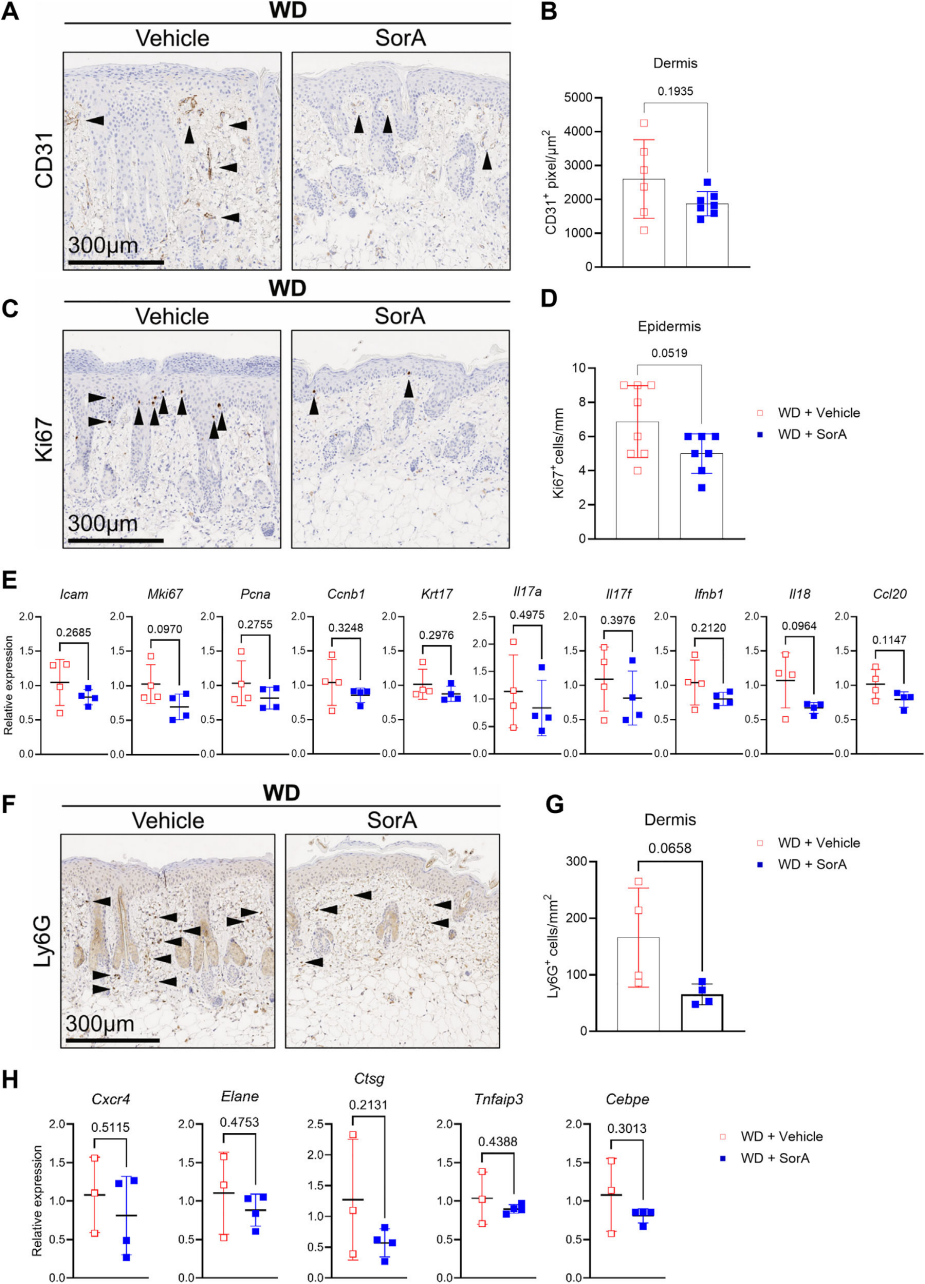
Prophylactic SorA administration is associated with modest differences in selected vascular, proliferative, and neutrophil‐associated skin readouts in Western diet (WD)‐fed imiquimod (IMQ)‐treated mice. Samples of IMQ‐treated back skin were collected on day 5 of the IMQ model from mice fed a Western diet (WD) for RNA extraction and immunohistochemical staining. (**A**) Representative immunohistochemical images of CD31. Black arrows indicate CD31^+^ cells in the dermis. (**B**) Quantification of CD31‐positive area in the dermis. (**C**) Representative immunohistochemical images of Ki67; black arrows indicate Ki67^+^ cells in the epidermis. (**D**) Quantified Ki67^+^ cells in the epidermis. (**E**) Real‐time PCR analysis of genes related to psoriatic inflammation. (**F**) Representative immunohistochemical staining of Ly6G. (**G**) Ly6G^+^ cell density in the dermis. (H) Real‐time PCR analysis of genes related to neutrophils in psoriatic inflammation. (**A**‐**B**) WD + vehicle = mice fed with WD and treated with vehicle (*n* = 6), and WD + SorA = mice fed with WD and treated with SorA (*n* = 7). (**C‐D**) WD + vehicle = mice fed with WD and treated with vehicle (*n* = 8), and WD + SorA = mice fed with WD and treated with SorA (*n* = 7). (**E**‐**G**) WD + vehicle = mice fed with WD and treated with vehicle (*n* = 4), and WD + SorA = mice fed with WD and treated with SorA (*n* = 4). (**H**) WD + vehicle = mice fed with WD and treated with vehicle (*n* = 3), and WD + SorA = mice fed with WD and treated with SorA (*n* = 4). Error bars represent standard deviation (SD). *p*‐values were obtained by an unpaired *t*‐test. ns, not significant = *p* > 0.05, *p < 0.05; **p < 0.01.

### 2.3 Prophylactic SorA Administration Leaves Hepatic Steatosis Scores Unchanged but is Associated With Reduced Hepatic Myeloid Cell Infiltration

WD contributes to MASLD by increasing fat accumulation, inflammation, and fibrosis in the liver. MASLD extent was evaluated using the nonalcoholic fatty liver disease activity score (NAS), a combined score of steatosis, inflammation, and ballooning. WD‐fed mice displayed hepatic steatosis and lobular inflammation without hepatocyte ballooning. Overall NAS did not differ between vehicle‐ and SorA‐treated mice (Figure 3A). We further evaluated the immune profile in the liver by flow cytometry (Figure 3B–E). Prophylactic SorA treatment showed no effect on hepatic T cell populations, including CD4^+^ Th, CD8^+^ cytotoxic (Tc), and γδ T cells (Figure 3C; Figure S3A–E). Additionally, type 17 T cells, including IL‐17A^+^ CD4^+^ T helper (Th17), IL‐17A^+^ CD8^+^ cytotoxic T (Tc17), and γδT17 cells, were unchanged (Figure 3D; Figure S3F). IL‐17A has been implicated in neutrophil recruitment and diet‐associated hepatocellular injury [28]. As prophylactic SorA treatment could influence early‐stage IL‐17A production in the IMQ model, we further evaluated neutrophil and monocyte recruitment in the livers of WD‐fed mice using flow cytometry and immunohistochemical staining. Prophylactic SorA treatment lowered neutrophil and monocyte proportions in the livers of WD‐fed IMQ‐treated mice (Figure 3E; Figure S3B,C). However, histological analysis of hepatic Ly6C showed no significant difference (data not shown). Furthermore, histological analysis revealed that prophylactic SorA treatment selectively reduced Ly6G^+^ neutrophil density, whereas MHCII^+^ cell density was unchanged in the livers of WD‐fed mice (Figure 3F,G). No pronounced systemic effects of SorA administration were observed in the spleen by flow cytometric analysis (Figure S4). No effect on IL‐17A‐expressing T cells was observed (Figure S4A), whereas neutrophils and monocytes were numerically lower without statistical significance (Figure S4B).

**FIGURE 3:**
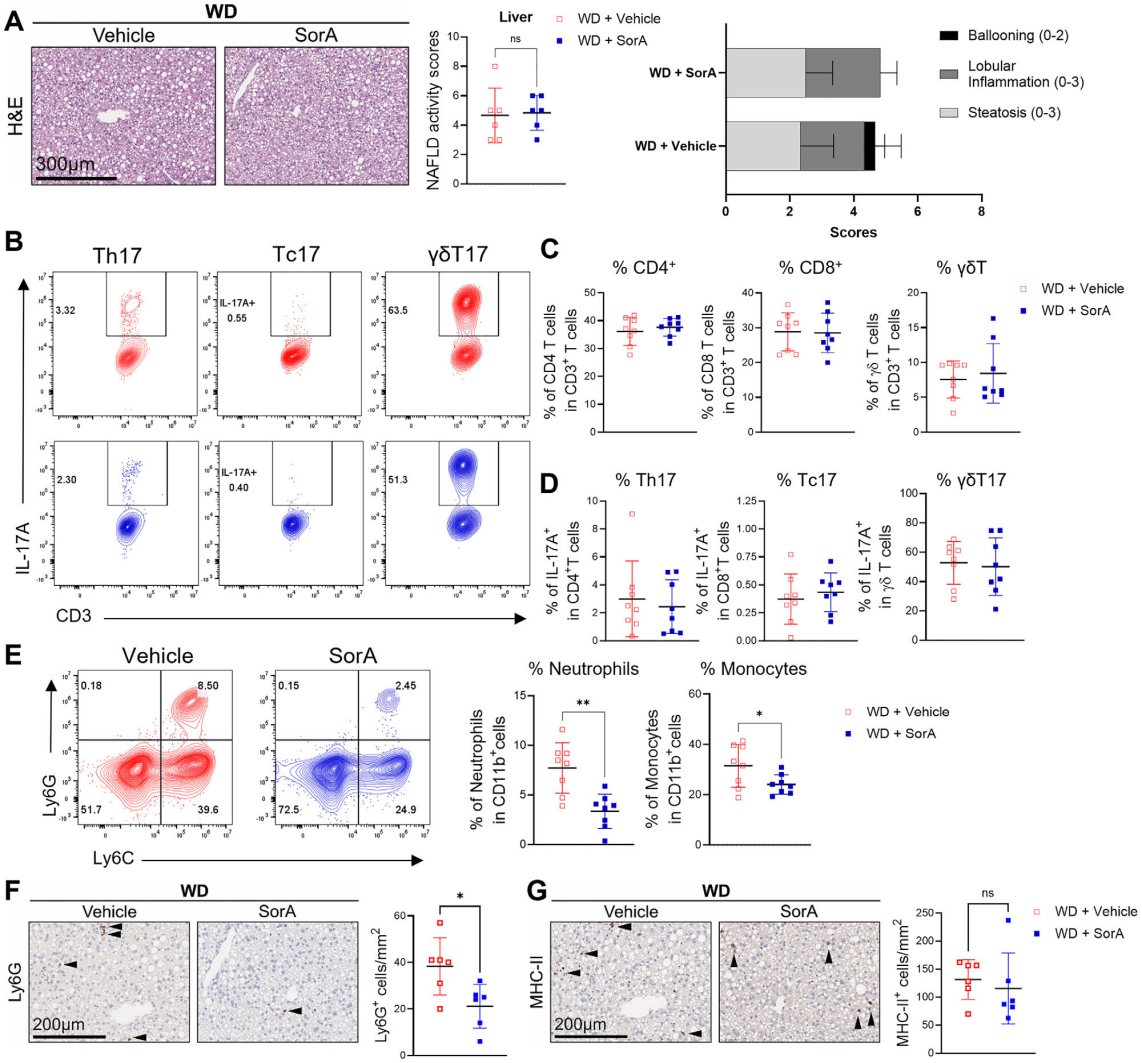
Prophylactic Soraphen A (SorA) is associated with reduced hepatic myeloid cell infiltration, while liver pathology scores remain unchanged in Western‐diet (WD)‐fed imiquimod (IMQ)‐treated mice. Liver samples were collected on day 5 of the IMQ model from WD‐fed mice for immunohistochemical staining and flow cytometry. (**A**) Nonalcoholic fatty liver disease (NAFLD) activity score (NAS) and its component scores, including steatosis, inflammation, and ballooning in liver sections with hematoxylin and eosin (H&E) staining. (**B**) Representative gating strategy of flow‐cytometric analysis for percentages of IL‐17A in CD4^+^ T helper (Th), CD8^+^ cytotoxic T (Tc), and gamma delta T (γδ T) cells. (**C**) Percentages of Th, Tc, and γδ T cells in the livers. (**D**) Percentages of IL‐17A^+^ cells among Th, Tc, and γδ T cells. (**E**) Percentages of neutrophils (CD11b^+^Ly6G^+^Ly6C^+^ cells) and monocytes (CD11b^+^Ly6G^−^Ly6C^+^ cells). (**F**) Ly6G^+^ neutrophil density in the liver. (**G**) MHCII^+^ cell density in the liver. (**A**, **F**, **G**) WD + vehicle = mice fed with WD and treated with vehicle (*n* = 6), and WD + SorA = mice fed with WD and treated with SorA (*n* = 6). (**B**–**E**) WD + vehicle = mice fed with WD and treated with vehicle (*n* = 8), and WD + SorA = mice fed with WD and treated with SorA (*n* = 8). Error bars represent standard deviation (SD). *p‐*values were obtained by unpaired *t*‐test. ns, not significant = *p* > 0.05, *p < 0.05; **p < 0.01.

### 2.4 Prophylactic SorA Administration Reduces IL‐17A^+^ CD4^+^ and CD8^+^ T Cells in Skin‐Draining Lymph Nodes

We next investigated skin‐draining lymph nodes via flow cytometry analysis to gain a better understanding of skin‐specific inflammation. Overall percentages and cell counts of Th, Tc, and γδ T cells were largely unchanged by SorA administration (Figure 4A). We further assessed the frequencies and absolute numbers of IL‐17A‐producing T cells in the skin‐draining lymph nodes linked to IMQ‐treated skin (Figure 4B–D). Specifically, SorA administration reduced the frequencies (Figure 4C) and absolute numbers of IL‐17A^+^ Th and Tc cells (Figure 4D), whereas γδT17 cells did not differ significantly in skin‐draining lymph nodes (Figure 4D). An imbalance between Treg and type 17 T cells is associated with higher psoriasis severity [29]. We previously showed that T cell‐specific ACC1 deficiency increased Treg/Th17 and Treg/Tc17 ratios in skin‐draining lymph nodes of the IMQ model [14]. Here, SorA‐treated WD‐fed mice had higher Treg/Th17 and Treg/Tc17 ratios than vehicle‐treated WD‐fed mice (Figure 4E).

**FIGURE 4:**
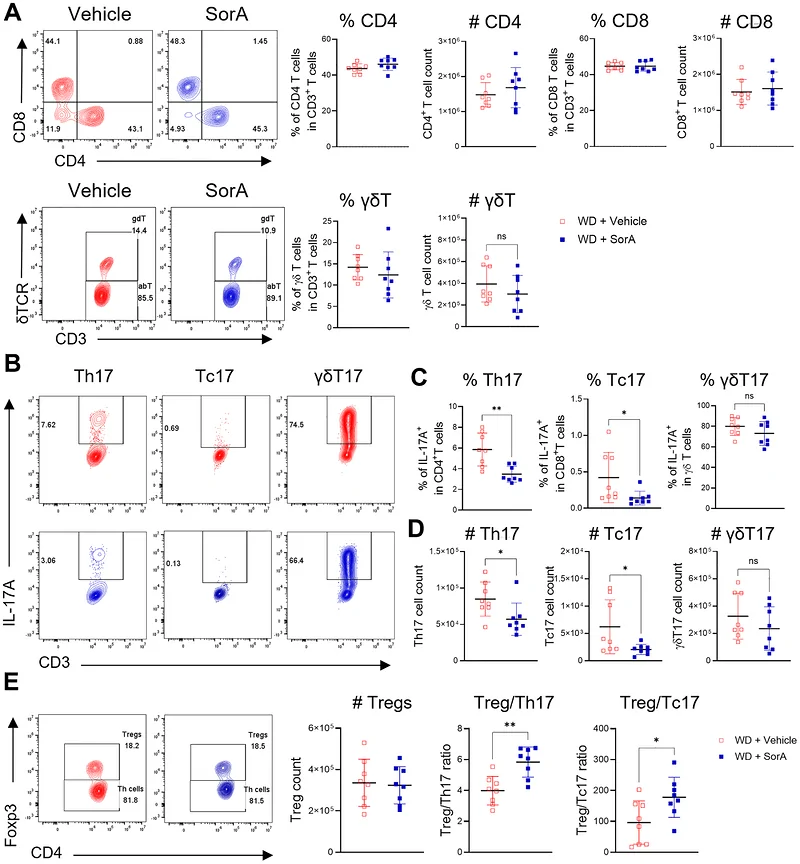
Prophylactic Soraphen A (SorA) administration reduces IL‐17A^+^ CD4^+^ and CD8^+^ T cells in skin‐draining lymph nodes from Western diet (WD)‐fed mice after imiquimod (IMQ)‐induced psoriasis‐like dermatitis. Skin‐draining lymph node (LN) samples were collected on day 5 of the IMQ model from WD‐fed mice for flow cytometric analysis. (**A**) Representative gating strategy for flow cytometry analysis for percentages and cell counts of CD4^+^ T helper (Th), CD8^+^ cytotoxic T (Tc), and γδ T cells in skin‐draining LNs. (**B**) Representative gating strategy for flow cytometry analysis of percentages of IL‐17A in CD4^+^ T helper (Th), CD8^+^ cytotoxic T (Tc), and γδ T cells. (**C**) Frequencies and (**D**) absolute numbers of IL‐17A^+^ Th17, Tc17, and γδT17 cells in skin‐draining LNs are shown. (**E**) Representative gating strategy of flow cytometry analysis for cell counts of Treg (CD3^+^CD4^+^Foxp3^+^) and Treg/Th17 and Treg/Tc17 ratios in the skin‐draining LNs during IMQ‐mediated inflammation is shown. Absolute cell counts were used to calculate the ratios. WD + vehicle = mice fed with WD and treated with vehicle (*n* = 8), and WD + SorA = mice fed with WD and treated with SorA (*n* = 8). Error bars represent standard deviation (SD). *p*‐values were obtained by an unpaired *t*‐test. ns, not significant = *p* > 0.05, *p < 0.05; **p < 0.01.

## 3 Discussion

In this study, we used a short prophylactic SorA regimen as an *in vivo* follow‐up to our previous mechanistic work on ACC1/SorA biology [7, 9]. Earlier studies established that SorA restrains Th17 differentiation, and that these effects are largely ACC1‐dependent [7, 9]. Furthermore, our work demonstrated that ACC1 deletion in T cells ameliorated psoriasiform phenotype and that ACC1 supports γδT17 effector function under psoriatic conditions [13, 14].

ACC1 and ACC2 have distinct metabolic functions that may differentially influence inflammatory responses. ACC1‐derived malonyl‐CoA fuels *de novo* FAS via fatty acid synthase (FASN), whereas ACC2‐derived malonyl‐CoA regulates mitochondrial fatty acid oxidation by inhibiting CPT1 [15]. Accordingly, ACC1 loss primarily impairs lipogenesis, while ACC2 deficiency enhances fatty acid oxidation and strongly affects obesity‐related metabolic phenotypes [30, 31, 32]. The ACC inhibitor SorA inhibits both isoforms [33]. However, the main effect of SorA on Th17 cells appears to be ACC1‐dependent [7, 16, 17].

Under ND conditions, systemic prophylactic SorA treatment did not measurably alter the severity of IMQ‐induced skin inflammation. One possible explanation for the absent ND effect is the transient pharmacological exposure in the present study compared with continuous T cell‐specific genetic deletion investigated in previous studies [13, 14], together with lower pre‐existing IL‐17‐driven inflammation under ND conditions. Additionally, we cannot rule out that ACC1‐dependent FAS is less limiting under ND conditions before psoriasis development. We therefore evaluated SorA in a WD‐exacerbated psoriasis model, in which metabolic inflammation is increased, because WD aggravates murine psoriatic dermatitis, partly by enhancing Th17 and γδT17 responses [3, 4, 34, 35, 36, 37, 38]. In line with the literature, WD increased body weight [39]. Based on the measured readouts, short‐term systemic SorA administration was tolerated and did not produce major changes in gross splenic immune cell composition. However, the present study was not designed as a formal toxicity assessment, and these data should therefore be interpreted as evidence of tolerability within the analyzed parameters rather than the absence of side effects. Interestingly, SorA administration slightly attenuated the well‐established body weight loss induced by IMQ application as a sign of systemic inflammation [40], in WD conditions, which is consistent with, but not sufficient to establish, a systemic anti‐inflammatory effect. This observation is relevant because total ACC1 depletion is lethal, underscoring the essential physiological role of ACC1 [41]. Importantly, as the peak of IMQ‐induced skin inflammation occurs between days 3 and 5, maintaining SorA exposure during the inflammatory phase might produce a larger effect; this remains to be investigated.

In line with our previous findings showing that ACC1 deletion in αβ T cells attenuates Th17 and Tc17 cell populations [14], SorA treatment significantly reduced Th17 and Tc17 cells, and increased Treg/Th17 and Treg/Tc17 ratios in skin‐draining LNs of WD‐fed IMQ‐treated mice. In contrast, γδT17 cells were not substantially affected by SorA treatment. Unlike Th17 and Tc17 cells, which differentiate from naïve T cells upon activation, γδ T cells constitute a largely tissue‐resident, innate‐like lymphocyte population [42, 43]. Their precommitted effector phenotype may confer a distinct metabolic flexibility, which could render them less susceptible to short‐term SorA exposure. Further studies are needed to determine whether this differential response reflects intrinsic metabolic differences or the short treatment window. The translational significance of the higher Treg/Th17 and Treg/Tc17 ratios in skin‐draining LNs also remains to be established.

Surprisingly, the most pronounced effects of SorA were observed on neutrophils. A polyunsaturated fatty acid‐rich high‐fat diet has been reported to promote neutrophil accumulation in the spleen and liver [44]. Additionally, FAS maintains neutrophil viability by sustaining membrane phospholipids [45]. Inducible FASN knockout mice were susceptible to neutropenic sepsis [45]. However, the role of FAS in neutrophils under psoriasis remains unclear. Here, the clearest SorA‐associated changes were observed in the myeloid compartment, particularly in hepatic neutrophils and monocytes, whereas skin neutrophil readouts were numerically lower but did not differ significantly. Furthermore, *Il18* transcript abundance was numerically lower in skin from WD‐fed mice receiving SorA but did not differ statistically from vehicle controls. IL‐18 is elevated in psoriatic skin lesions from patients [46] and contributes to neutrophil microabscess formation in the IMQ model [47]. Because IL‐18 can be produced by myeloid cells, including neutrophils [48], a link between SorA, neutrophils, and IL‐18 is conceivable but remains speculative in the present study. The short prophylactic regimen may have produced differential effects across tissues and cell compartments, but the present design cannot resolve cell‐intrinsic sensitivity.

Psoriasis is strongly associated with endothelial dysfunction, characterized by enhanced leukocyte adhesion, vascular hyperpermeability, and altered endothelial homeostasis [49]. Pro‐inflammatory cytokine signaling can activate endothelial cells and alter adhesion‐molecule and chemokine expression, thereby influencing leukocyte recruitment to inflamed skin [49, 50]. In our study, reduced erythema along with lower CD31 and ICAM‐1 readouts under SorA is compatible with a vascular contribution, although these data are descriptive and not mechanistic. A central component of vascular remodeling during psoriasis is EC migration, dependent on filopodia formation [18]. Human ECs isolated from psoriasis patients display significantly enhanced migratory and angiogenic capacity compared with those from healthy controls [51]. Inhibition of ACC1 was shown to downregulate EC filopodia formation and abrogate EC migration, linking lipid metabolism, specifically FAS, to EC motility [18]. Hence, inhibition of ACC1 by SorA may alter filopodia formation and, consequently, limit aberrant EC migration in psoriasis. This may contribute to the observed reduction of IMQ‐induced skin inflammation and erythema in our murine model.

FAS is required for skin barrier function [52]. Recent studies have shown that inhibiting acetyl‐CoA synthesis by targeting the genes encoding *Acly* and *Acss2* in a keratinocyte‐specific manner induces skin barrier dysfunction by limiting FAS, triggering epidermal hyperplasia [53]. SorA treatment could theoretically produce similar effects by limiting the malonyl‐CoA required for FAS. Although we observed a reduction in epidermal thickness within our model, skin barrier effects may counterbalance the anti‐inflammatory effect of SorA in the context of psoriasis.

Further, the steatotic liver phenotype remained overall unchanged except for reduced myeloid infiltration under SorA. Additional qPCR and histological readouts of macrophage‐associated polarization, proliferation, and inflammation showed no consistent SorA‐associated differences (data not shown). This contrasts with reports that liver‐specific ACC1 deletion reduces hepatic triglyceride accumulation without markedly altering glucose homeostasis [54]. Previous work using 50 or 100 mg/kg/day SorA in high‐fat‐fed mice reported reductions in body weight and selected metabolic endpoints [55]. The short‐term prophylactic administration of SorA in this study is likely insufficient to reverse 14 weeks of WD‐induced MASLD. The myeloid changes may therefore represent an earlier or more persistent response to the prophylactic regimen, but the current data do not define the underlying mechanism [56]. Direct effects of SorA on neutrophils cannot be excluded.

In line with the seminal protocol by van der Fits et al. [40], we used female mice in this study. Sex‐dependent effects of both the IMQ model (female mice develop a more severe disease than male mice within this model [57, 58]) and diet (female mice may show a slower or less pronounced response to high‐calorie diets [39, 59]) must be considered. Because only female mice were studied, generalizability to male mice remains uncertain.

Taken together, our findings support a context‐dependent effect of short‐term prophylactic SorA in WD‐exacerbated IMQ‐induced psoriasiform inflammation. The clearest changes were observed in IL‐17A^+^ CD4^+^ and CD8^+^ T cells of skin‐draining lymph nodes, hepatic myeloid infiltration, and selected skin readouts, whereas effects on γδT17 cells and several skin molecular markers were modest. These data position the study as an *in vivo* proof‐of‐concept for ACC‐targeted immunometabolic modulation in a metabolically aggravated setting.

### 3.1 Data Limitations and Perspectives

The main limitations of this study are the prophylactic design before psoriasiform dermatitis induction, the short duration of SorA exposure, and the use of SorA as a dual ACC1/2 inhibitor. Accordingly, the present data should not be interpreted as evidence of therapeutic efficacy in established disease or as definitive proof of ACC1‐specific action *in vivo*. Rather, they provide a rationale for further testing of treatment timing, dose, and tissue‐specific target engagement in WD‐associated psoriasiform inflammation. These findings lay the basis for further preclinical studies of ACC‐targeted immunometabolic modulation in metabolically aggravated psoriasiform inflammation.

## 4 Methods

### 4.1 Mice

Experiments were approved by the Animal Care and Use Committee of the Land of Rhineland‐Palatine (approval no. G23‐1‐020). Guidelines from Directive 2010/63/EU of the European Parliament on the protection of animals used for scientific purposes were followed. The mice were housed in the animal facility of the University Medical Center of the Johannes Gutenberg University of Mainz under specified pathogen‐free conditions. Female mice were used in alignment with the original protocol by van der Fits et al. [40]. and our previous work [13, 14], and due to the fact that their hair‐cycle characteristics facilitate repeated topical application [60, 61].

### 4.2 Diet‐Exacerbated Psoriasis Mouse Model and Soraphen A (SorA) Administration

Seven‐week‐old C57BL/6J (Janvier Labs) female mice were fed a normal diet (ND) or a Western diet (WD; 40% kcal fat, 20% kcal fructose, 2% cholesterol; Research Diets, #D16022301i) for a total of 14 weeks. Soraphen A (SorA) compound is poorly water‐soluble and was reconstituted in corn oil and then mixed with the lecithin solution to form an emulsion for intraperitoneal (i.p.) administration, as described previously [62]. In the group of mice that received SorA, each mouse received 20 mg/kg SorA daily, as previously published [62]. To assess the effect of prophylactic SorA administration, mice were first maintained on ND or WD for 12 weeks and then received SorA or vehicle intraperitoneally once daily for 10 consecutive days before IMQ challenge. Psoriasiform dermatitis was subsequently induced by topical IMQ for 5 days, after which tissues were collected for analysis. The topical IMQ‐induced psoriasis model was performed as described previously [14]. Briefly, C57BL/6J mice were shaved and depilated with hair removal cream (Veet, Reckitt Benckiser Group, Slough, England) on day ‐2 of the IMQ model. After 2 days, mice were topically treated with 60 mg Aldara (5% IMQ, Meda, Solna, Sweden; 50 mg on the back skin and 5 mg per ear) for 5 consecutive days. Psoriasis‐like dermatitis was assessed using a modified Psoriasis Area and Severity Index (PASI), as described previously [14, 40].

### 4.3 Histology

Tissue samples were fixed in 4% paraformaldehyde solution (PFA, Carl Roth GmbH+Co.KG, Karlsruhe, Germany), dehydrated, and embedded in paraffin. These PFA‐fixed, paraffin‐embedded blocks were sectioned at 2 µm and stained with H&E (Morphisto, Germany), CD31 (clone: SZ31, DIANOVA, #DIA‐310), Ki67 (clone SP6, Thermo Fisher Scientific, Massachusetts, USA; #RM‐9106‐S1), Ly6G (clone: 1A8, BD Pharmingen, #551459), and MHCII (clone: M5/114.15.2, Novus Biologicals, # NBP1‐43312). For staining, sections were deparaffinized, rehydrated, and antigens were retrieved with Bond citrate solution (AR9961, Leica Biosystems Nussloch GmbH, Nussloch, Germany), Bond EDTA solution (AR9640, Leica Biosystems Nussloch GmbH), or Bond proteolytic enzyme kit (AR9551, Leica Biosystems Nussloch GmbH). Sections were incubated in Bond Primary Antibody Diluent (Leica Biosystems Nussloch GmbH), and staining was performed on a BOND‐MAX Automated IHC/ISH Stainer (Leica Biosystems Nussloch GmbH). Slides were scanned using an Aperio AT2 scanner (Leica Biosystems Nussloch GmbH) at 20x and analyzed with Aperio ImageScope (ver. 12.4.0.5043, Leica) and Fiji (ImageJ ver. 1.52e, National Institutes of Health, USA). The non‐alcoholic fatty liver disease activity score (NAS) [63] was assessed by a board‐certified pathologist (FKFK) blinded to the treatment group.

### 4.4 Isolation of RNA and Quantitative Real‐Time PCR

Bulk RNA was extracted in 1 mL QIAzol (Qiagen, Hilden, Germany) using the RNeasy Mini Kit (Qiagen). After quantity and quality control were performed with the NanoDrop analyzer (Thermo Fisher Scientific), 1 µg of purified RNA was subsequently reverse‐transcribed into cDNA using the high‐capacity cDNA reverse transcription kit (Applied Biosystems, Massachusetts, USA) according to the manufacturer's instructions. Quantitative real‐time PCR was performed in duplicate in 384‐well plates using Fast Start SYBR Green Master Rox (Roche, Basel, Switzerland) on a QuantStudio 5 real‐time PCR system (Applied Biosystems) using primer pairs provided in Table 1. Relative expression was calculated using the 2^−ΔΔC^ *^t^* method, with Rps14 as the reference gene and the WD + vehicle group as calibrator. qPCR data are presented as relative expression, as indicated in the figure legends, and significance was tested by an unpaired *t*‐test.

**TABLE 1:** Primers for qPCR analysis.

Target gene name	Primer sequence	
*Rps14*	Forward	5’‐GAC CAA GAC CCC TGG ACC T‐3’
	Reverse	5’‐CCC CTT TTC TTC GAG TGC TA‐3’
*Il17a*	Forward	5’‐CAG ACT ACC TCA ACC GTT CCA C‐3’
	Reverse	5’‐TCC AGC TTT CCC TCC GCA TTG A‐3’
*Il17f*	Forward	5’‐AAC CAG GGC ATT TCT GTC CCA C‐3'
	Reverse	5’‐GGC ATT GAT GCA GCC TGA GTG T‐3’
*Ifnb1*	Forward	5’‐ATG AAC AAC AGG TGG ATC CTC C‐3’
	Reverse	5’‐AGG AGC TCC TGA CAT TTC CGA A‐3’
*Il18*	Forward	5’‐TGA CCA AGT TCT CTT CGT TG‐3’
	Reverse	5’‐GGT TCA CTG GCA CTT TGA‐3’
*Ccl20*	Forward	5’‐TTT TGG GAT GGA ATT GGA CAC‐3’
	Reverse	5’‐TGC AGG TGA AGC CTT CAA CC‐3’
*Keratin 17 (Krt17)*	Forward	5’‐GCC CAC CTG ACT CAG TAC AA‐3’
	Reverse	5’‐GGA GCT GAG TCC TTA ACG GG‐3’
*Mki67*	Forward	5’‐GAG GAG AAA CGC CAA CCA AGA G‐3’
	Reverse	5’‐TTT GTC CTC GGT GGC GTT ATC C‐3’
*Pcna*	Forward	5’‐CAA GTG GAG AGC TTG GCA ATG G‐3’
	Reverse	5’‐GCA AAC GTT AGG TGA ACA GGC TC‐3’
*Ccnb1*	Forward	5’‐AGA GGT GGA ACT TGC TGA GCC T‐3’
	Reverse	5’‐GCA CAT CCA GAT GTT TCC ATC GG‐3’
*Icam1*	Forward	5’‐ACT GCT TGG GGA ACT GGA C‐3’
	Reverse	5’‐AGG CAT GGC ACA CGT ATG TA‐3’
*Cxcr4*	Forward	5’‐AAA CCT CTG AGG CGT TTG GT‐3’
	Reverse	5’‐AGC AGG GTT CCT TGT TGG AG‐3’
*Elane*	Forward	5’‐CAG GAA CTT CGT CAT GTC AGC AG‐3’
	Reverse	5’‐AGC CAT TCT CGA AGA TCC GCT G‐3’
*Ctsg*	Forward	5’‐AGT CCA GAA GGG CTG AGT GCT T‐3’
	Reverse	5’‐GCA CTG TGA TGA GTT GCT GGG T‐3’
*Tnfaip3*	Forward	5’‐GAA CAG CGA TCA GGC CAG G‐3’
	Reverse	5’‐GGA CAG TTG GGT GTC TCA CAT T‐3’
*Cebpe*	Forward	5’‐GCA GCC ACT TGA GTT CTC AGG‐3’
	Reverse	5’‐GAT GTA GGC GGA GAG GTC GAT‐3’

### 4.5 Flow Cytometry Analysis

Single‐cell suspensions were prepared from liver, spleen, and skin‐draining lymph nodes. Spleen and lymph nodes were dissociated by passing through a 70 µm mesh strainer using a 5 mL syringe plunger. Liver samples were cut into small pieces, followed by enzymatic digestion for 45 min at 37°C with 0.5 mg·mL^−1^ collagenase IV (Sigma‐Aldrich) and 50 U·mL^−1^ DNase I (Applichem). Hepatic leukocytes were isolated by Percoll density gradient centrifugation. Single‐cell suspensions were incubated with an in‐house Fc receptor blocking reagent. Dead cells were excluded using the LIVE/DEAD fixable aqua dead cell stain kit (Invitrogen, Carlsbad, CA, USA). For analysis of surface markers, cells were stained in PBS containing 0.25% BSA (Roche, Mannheim, Germany) and 0.02% NaN3 (Carl Roth GmbH+Co.KG) using the following fluorescence‐conjugated monoclonal antibodies: CD3ε (145‐2C11; eBiosciences), CD8α (Ly2; eBiosciences), CD45 (30‐F11; BioLegend, San Diego, CA), CD4 (GK1.5; eBiosciences), Ly6C (HK1.4; BioLegend), Ly6G (1A8; BioLegend), CD11b (M1/70; BioLegend), and TCRδ (GL3; eBiosciences). For intracellular cytokine and transcription factor staining, cells were stained with IL‐17A (eBio17B7; eBiosciences) and Foxp3 (FJK‐16sl; eBiosciences) using the Foxp3/transcription factor fixation/permeabilization kit (eBiosciences) according to the manufacturer's instructions. For cytokine staining, cells were stimulated *in vitro* in the presence of phorbol‐12‐myristate‐13‐acetate (0.1 µg·mL^−1^; Sigma‐Aldrich) and ionomycin (1 µg·mL^−1^; Sigma‐Aldrich) for 2 h, followed by incubation for 2 h with brefeldin A (5 µg·mL−1; eBiosciences) before staining. Cells were acquired on CytoFLEX S (Beckman Coulter) or Cytek Northern Lights (Cytek Biosciences), and data were analyzed with FlowJo software (v.10.8.1, Tree Star). Absolute cell numbers were obtained by total viable‐cell counting and volumetric calculation.

### 4.6 Statistical Analysis

Statistical analysis was performed using GraphPad Prism version 9/10. Data are shown as mean ± standard deviation (SD) unless stated otherwise. Clinical scores measured over time were analyzed by two‐way ANOVA or mixed‐effects/repeated‐measures models, as indicated in the figure legends. Two‐group endpoint comparisons were analyzed using two‐sided unpaired *t*‐tests. A *p*‐value < 0.05 was considered statistically significant.

## Author Contributions

Y.S.K., K.S.K., T.B., M.H., and T.S. designed the study. Y.S.K., K.S.K., T.B., G.J.G., A.L.K., and F.B. performed the experiments. F.K.F.K. performed the blinded NAS assessment. D.H. performed immunohistochemical staining. S.H.K. and R.M. synthesized and provided Soraphen A and contributed to discussions on formulation, application, and interpretation of the results. Y.S.K., K.S.K., T.B., M.H., and T.S. wrote the first draft of the manuscript. M.H. and T.S. supervised the study. All authors contributed to the review and editing of the final manuscript.

## Ethics Statement

Experiments were approved by the Animal Care and Use Committee of the Land of Rhineland‐Palatinate (approval no. G23‐1‐020).

## Conflicts of Interest

The authors declare no conflicts of interest.

## Supporting information




**Supporting File**: eji70298‐sup‐0001‐SuppMat.pdf.

## Data Availability

All data are available from the corresponding authors upon reasonable request.
