# Supplementary material for: Prophylactic Soraphen A Treatment Attenuates Western Diet‐Exacerbated IMQ‐Induced Psoriasiform Inflammation
Source: Eur J Immunol. 2026 Oct 27;56(10):e70298. doi: 10.1002/eji.70298 (PMC13714554; doi:10.1002/eji.70298)
Supplement: Supplementary file 1 [file EJI-56-e70298-s001.pdf]

Supplementary Figures

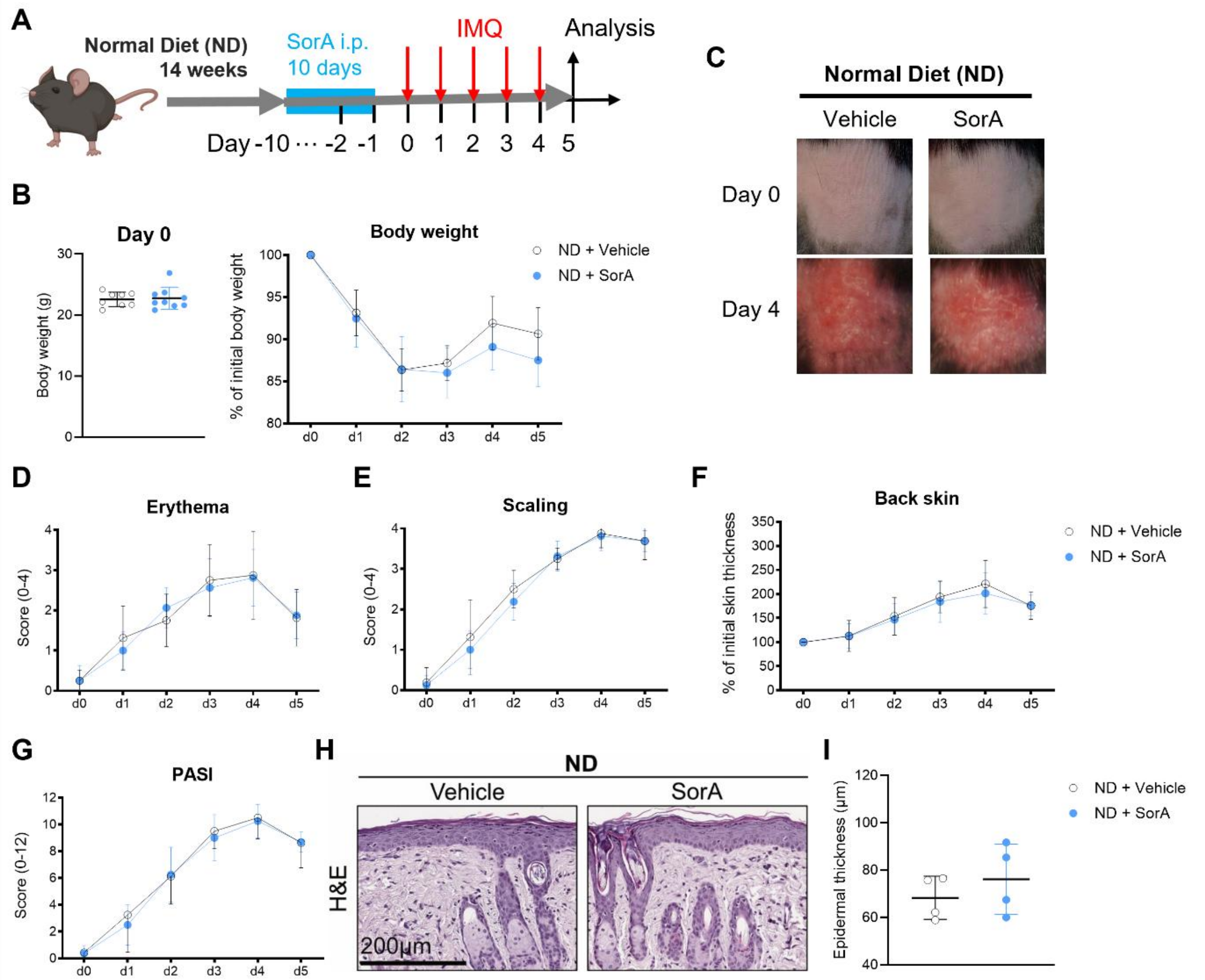

Supplementary Figure 1. Prophylactic Soraphen A (SorA) administration shows no effect on Imiquimod (IMQ)-induced psoriasis-like dermatitis under normal diet conditions. (**A**) 7-week-old female mice were fed a normal chow diet (ND) for 12 weeks, followed by 10 daily intraperitoneal injections of the ACC inhibitor Soraphen A (SorA; 20 mg/kg/day) or vehicle, and five days of topical imiquimod (IMQ) challenge (total study duration approximately 14 weeks). (**B**) Body weight on day 0 (= start of IMQ treatment) and body-weight change during IMQ treatment. (**C**) Representative images of Vehicle and SorA-treated mice on day 0 and 4 of IMQ application. Clinical scores of (**D**) erythema, (**E**) scaling, (**F**) thickness of IMQ-treated back skin, and (**G**) cumulative Psoriasis Area and Severity Index (PASI) Score (Erythema, Scaling, and Back skin thickness). Skin sections were collected from the IMQ-treated back skin area on day 5 for hematoxylin and eosin (H&E) staining, and representative histological sections are shown in (**H)** and quantified in (**I**). (**A**-**G**) ND + vehicle = mice fed with ND and treated with vehicle (n=8), ND + SorA = mice fed with ND and treated with SorA (n=8). (**I**) ND + vehicle (n=4), ND + SorA (n=4). Error bars represent standard deviation (SD). p-values were obtained by two-way ANOVA in (**B-G)** and an unpaired t-test in (**I**). ns, not significant = p > 0.05

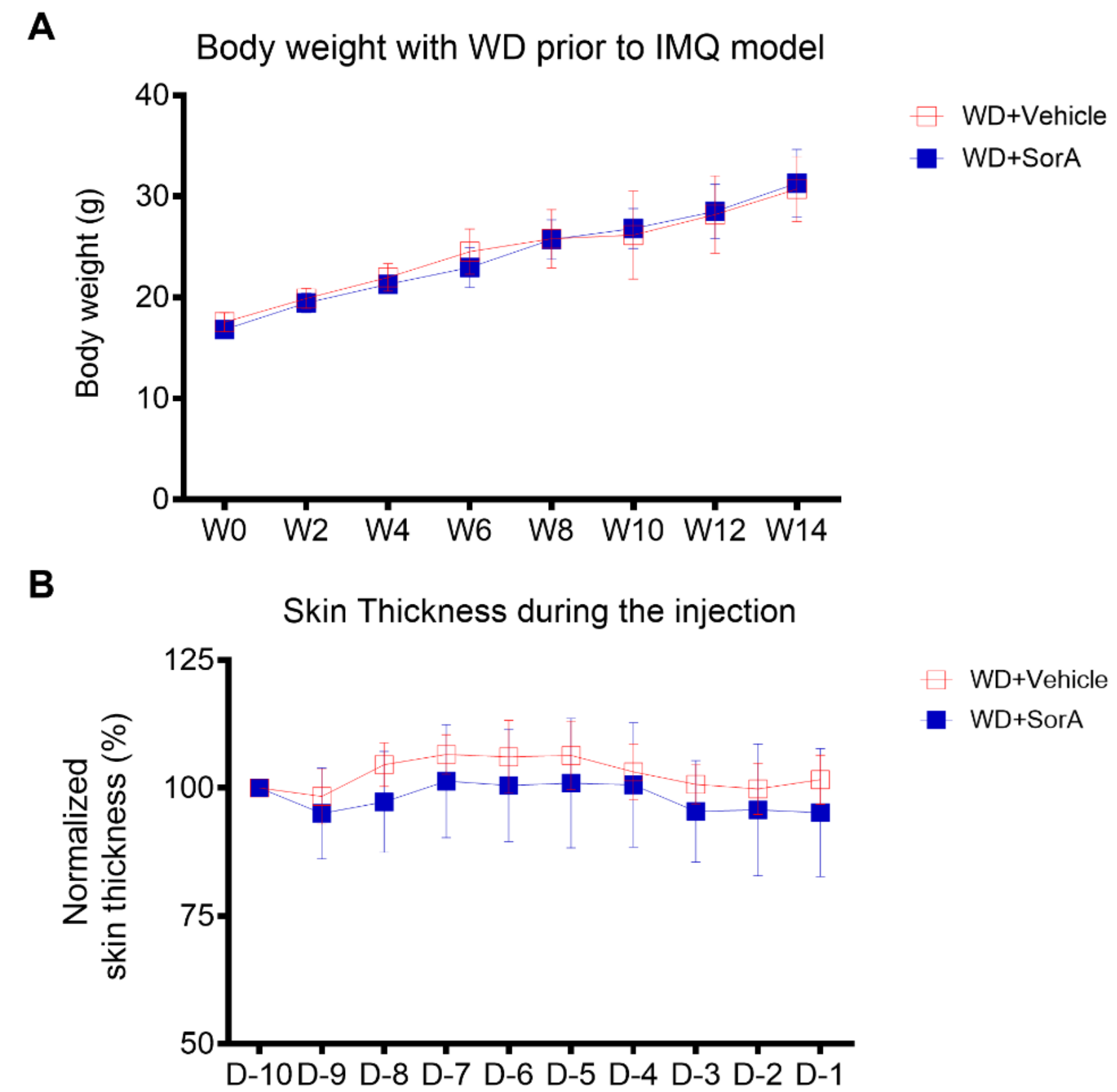

Supplementary Figure 2. Prophylactic Soraphen A (SorA) administration in the Western diet (WD)-fed mice. (**A**) Body weight in grams (g) prior to Imiquimod application over 14 weeks of Western diet (WD) feeding in vehicle and Soraphen A (SorA) cohorts. (**B**) Skin thickness during injection of vehicle or SorA in WD-fed mice, normalized to baseline skin thickness. Days (D) -10 to -1 prior to Imiquimod application. WD + vehicle = mice fed with WD and treated with vehicle (n=4), and WD + SorA = mice fed with WD and treated with SorA (n=4). Error bars represent standard deviation (SD).

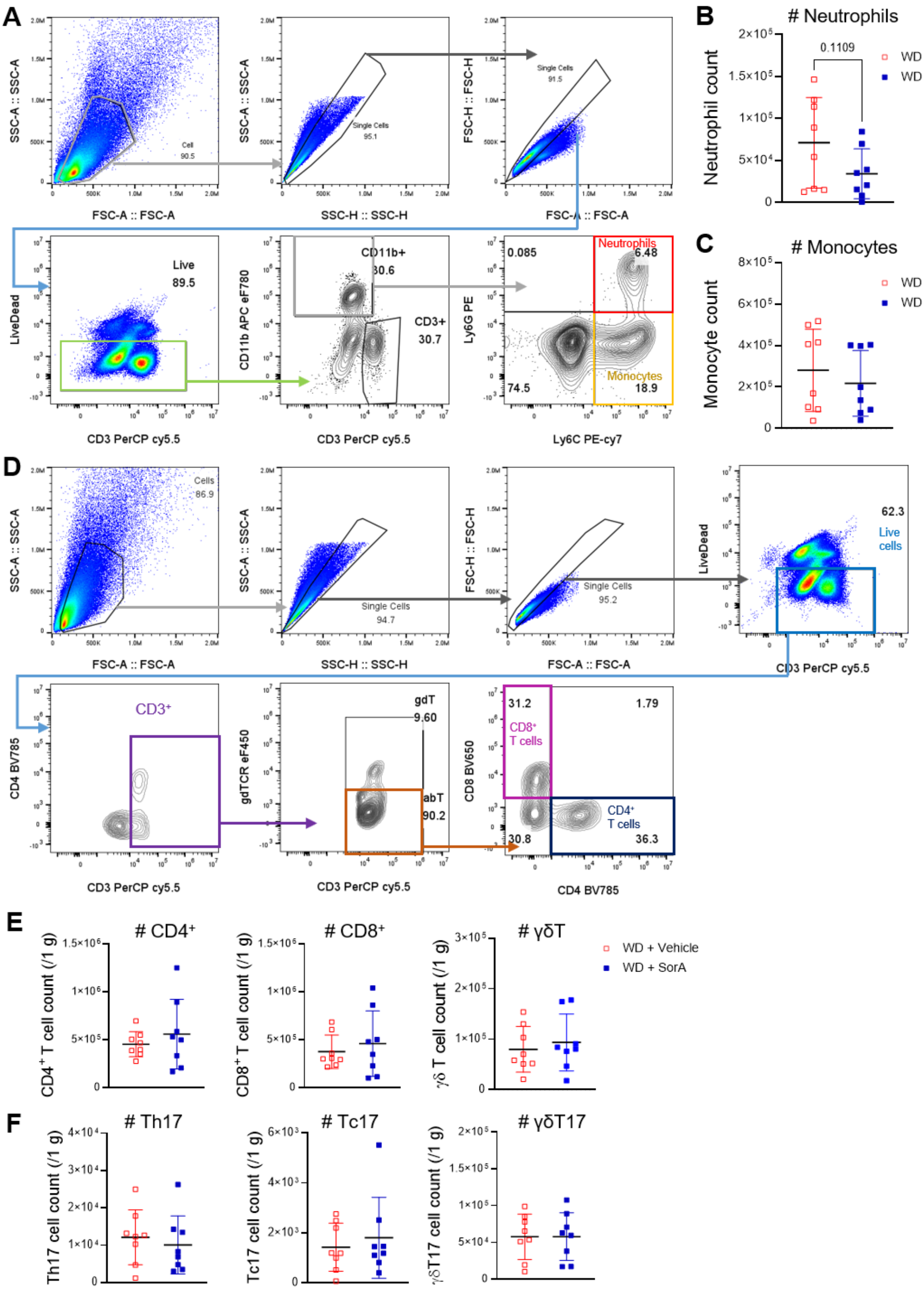

Supplementary Figure 3. Prophylactic Soraphen A (SorA) administration reduces hepatic neutrophil numbers by trend, while T cell counts remain unchanged after Imiquimod (IMQ)-induced psoriasis-like dermatitis in Western diet (WD)-fed mice. Liver samples were collected on day 5 of the IMQ model from WD-fed mice for flow cytometry. (**A**) Representative gating strategy of flow cytometry analysis for percentages and cell counts of neutrophils (**B**) and monocytes (**C**). (**D**) Representative gating strategy of flow cytometry analysis for cell counts of $CD4^+$ T helper (Th), $CD8^+$ cytotoxic T (Tc), and gamma delta T (γδ T) cells (**E**) and IL-17A+ Th17, Tc17, and γδT17 cells (**F**) in the liver. Error bars represent

standard deviation (SD). p-values were obtained by an unpaired t-test. ns, not significant = $p > 0.05$.

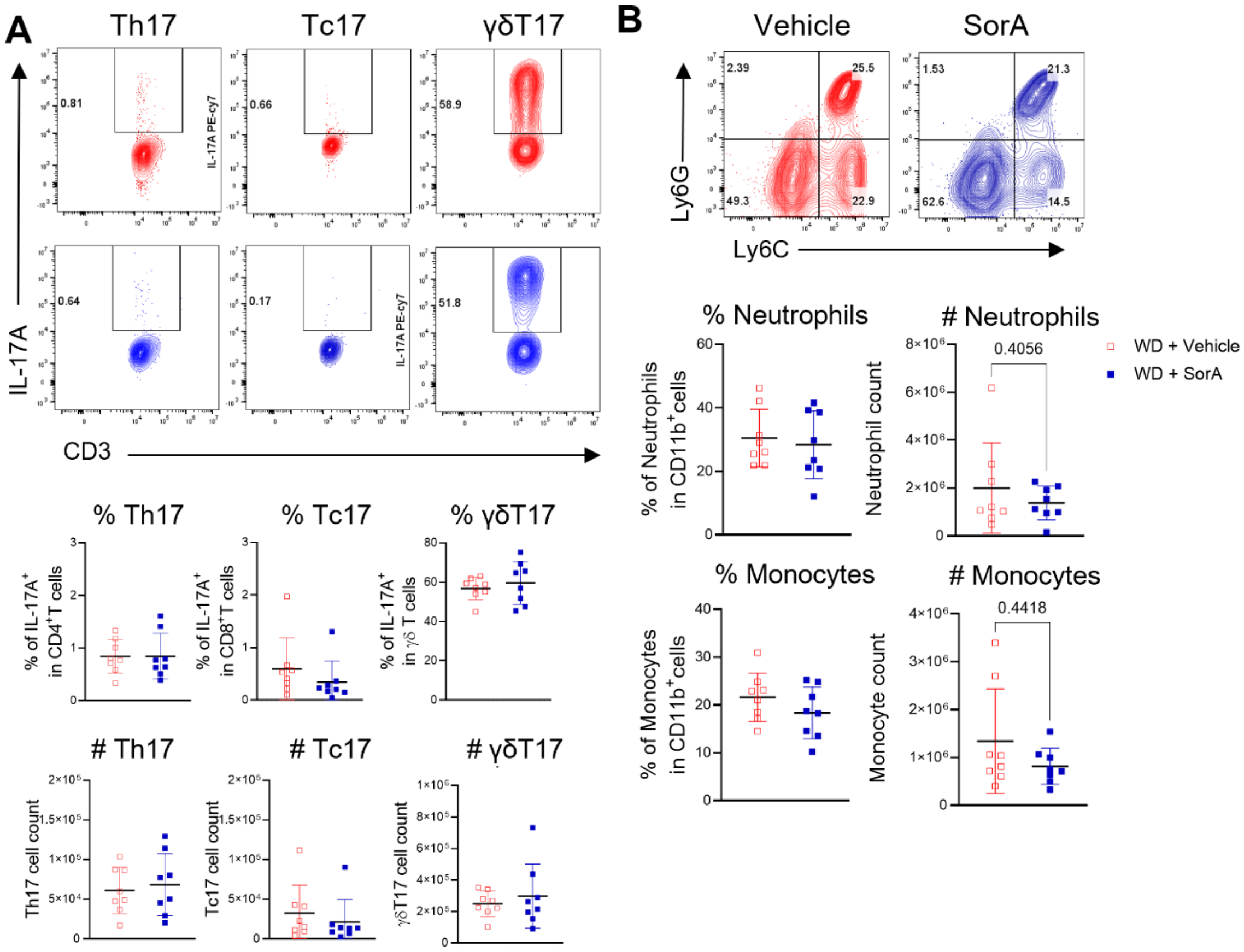

Supplementary Figure 4. Prophylactic Soraphen A (SorA) does not reveal major splenic immune changes after IMQ-induced psoriasiform dermatitis in Western-diet (WD)-fed mice. (**A**) Representative gating strategy of flow cytometry analysis for percentages and cell counts of IL-17A in $CD4^+$ T helper (Th), $CD8^+$ cytotoxic T (Tc), and gamma delta T (γδ T) cells in spleens. (**B**) Representative gating strategy of flow cytometry analysis for percentages and cell counts of neutrophils and monocytes. Error bars represent standard deviation (SD). *p*-values were obtained by an unpaired t-test. ns, not significant = $p > 0.05$
